# Risk factors for loss to follow-up in patients with gout: A Korean prospective cohort study

**DOI:** 10.1371/journal.pone.0318564

**Published:** 2025-02-07

**Authors:** Hyunsue Do, Chang-Nam Son, Hyo Jin Choi, Ji Hyoun Kim, Min Jung Kim, Kichul Shin, Sang-Hyon Kim, Byoongyong Choi, You-Jung Ha, Joong Kyong Ahn, Hyun-Ok Kim, Sung Won Lee, Chang Hoon Lee, Ran Song, Kyeong Min Son, Seung-Geun Lee, Ki Won Moon

**Affiliations:** 1 Division of Rheumatology, Department of Internal Medicine, Kangwon National University School of Medicine, Chuncheon, Gangwon-do, Republic of Korea; 2 Division of Rheumatology, Department of Internal Medicine, Eulji University School of Medicine, Uijeongbu, Gyeonggi-do, Republic of Korea; 3 Division of Rheumatology, Department of Internal Medicine, Gil Medical Center, Gachon University College of Medicine, Incheon, Republic of Korea; 4 Division of Rheumatology, Department of Internal Medicine, Chungbuk National University Hospital, Cheongju, Chungcheongbuk-do, Republic of Korea; 5 Division of Rheumatology, Department of Internal Medicine, Seoul Metropolitan Government-Seoul National University Boramae Medical Center, Seoul, Republic of Korea; 6 Division of Rheumatology, Department of Internal Medicine, Keimyung University School of Medicine, Daegu, Republic of Korea; 7 Division of Rheumatology, Department of Internal Medicine, Seoul Metropolitan Seoul Medical Center, Seoul, Republic of Korea; 8 Division of Rheumatology, Department of Internal Medicine, Seoul National University Bundang Hospital, Seongnam, Gyeonggi-do, Republic of Korea; 9 Division of Rheumatology, Department of Internal Medicine, Kangbuk Samsung Hospital, Sungkyunkwan University School of Medicine, Seoul, Republic of Korea; 10 Division of Rheumatology, Department of Internal Medicine, Gyeongsang National University School of Medicine, Changwon, Gyeongsangnam-do, Republic of Korea; 11 Division of Rheumatology, Department of Internal Medicine, Soon Chun Hyang University Hospital, Cheonan, Chungcheongnam-do, Republic of Korea; 12 Division of Rheumatology, Department of Internal Medicine, Wonkwang University School of Medicine, Iksan, Jeonbuk-do, Republic of Korea; 13 Division of Rheumatology, Department of Internal Medicine, Kyung Hee University College of Medicine, Seoul, Republic of Korea; 14 Division of Rheumatology, Department of Internal Medicine, Hallym University Dongtan Sacred Heart Hospital, Hwaseong-si, Gyeonggi-do, Republic of Korea; 15 Division of Rheumatology, Department of Internal Medicine, Pusan National University School of Medicine, Pusan National University Hospital, Busan, Republic of Korea; Straub Medical Center, UNITED STATES OF AMERICA

## Abstract

**Objectives:**

Gout, a common form of inflammatory arthritis, is often managed with urate-lowering therapy, but many patients only adhere to treatment during gout attacks, resulting in poor follow-up and suboptimal management. This study aimed to identify characteristics associated with loss to follow-up (LTFU) and develop strategies for better patient management.

**Methods:**

Data were analyzed from the Urate Lowering TheRApy in gout (ULTRA) registry, a prospective cohort of Korean gout patients recruited since September 2021. Patients aged 18 or older who met the 2015 ACR/EULAR classification criteria were included. Demographic data, clinical characteristics, lifestyle habits, comorbidities, and quality of life assessments using the Korean Gout Impact Scale (K-GIS) and EuroQol 5-Dimension (EQ-5D) were collected at baseline, six months, and annually. LTFU was defined as missing a clinic visit for more than a year. Logistic regression was used to determine factors associated with LTFU.

**Results:**

Among 269 patients, 125 (46.5%) were classified as LTFU. Patients not lost to follow-up experienced more frequent gout attacks (P = 0.020) and expressed greater concerns about future flares (P = 0.034). In contrast, LTFU patients had higher levels of anxiety (P = 0.049), depressive symptoms (P = 0.009), impaired mobility (P = 0.002), and a higher EQ-5D score (P = 0.002). Logistic regression identified frequent gout attacks, concerns about attacks, anxiety, impaired mobility, and EQ-5D scores as significant predictors of LTFU.

**Conclusion:**

Fewer gout attacks, heightened anxiety and depression, and lower quality of life were key factors associated with LTFU. Providing emotional support and comprehensive education may enhance follow-up and improve gout management.

## Introduction

Gout, a prevalent form of inflammatory arthritis, may potentially be more effectively controllable relative to other forms of arthritis [1]. Patients with gout are recommended to undergo long-term urate-lowering therapy (ULT) along with lifestyle modifications, particularly regarding their dietary habits [2, 3]. However, many patients with gout only take medication during acute attacks, and persistent follow-up visits are not well-maintained [4–6].

This has led to inadequate management and complications associated with gouty arthritis. Like other chronic diseases, gout requires consistent medication management and regular follow-up visits. If follow-up is lost and gout treatment is not consistently administered [7], patients may experience recurrent gout attacks that progress to chronic gouty arthritis [8, 9]. Despite this, awareness of the need for consistent gout management has been significantly underestimated. Many patients with gout perceive it as a condition requiring treatment only during acute attacks [9–11]. This misconception can result in inadequate long-term care and an increased risk of complications. Therefore, both patients and healthcare providers must understand the importance of continuous treatment and regular monitoring for appropriate management and prevention of disease progression. Educating patients about the natural course of gout and the necessity of ongoing urate-lowering therapy, even when symptoms are absent, is essential for improving outcomes and quality of life.

Although numerous studies have examined drug adherence in patients with gout, research on loss to follow-up (LTFU) remains limited. To the best of our knowledge, there has been only one previous study on LTFU within a gout cohort. Perez-Ruiz *et al*. reported that older age, high drug adherence, and consultation with primary care providers were independently associated with persistence during follow-up [12]. LTFU is an important clinical issue distinct from drug adherence. If we can identify individuals at high risk of LTFU during the initial visit, follow-up rates may be improved through appropriate education and intervention. Therefore, we aimed to investigate this issue in a Korean prospective cohort of patients with gout by determining which patients are more likely to experience LTFU, identifying associated risk factors, and evaluating the extent to which specific factors increase the risk of LTFU.

## Materials and methods

### Participants

We used data from the ‘Aftermath of Urate-Lowering Therapy in Gout (ULTRA)’ registry [13]. The ULTRA registry is a prospective cohort study of Korean patients with gout, conducted at multiple centers nationwide since September 2021, involving rheumatologists from major secondary and tertiary hospitals in both rural and urban areas of Korea.

Patients aged ≥18 years who fulfill the 2015 classification criteria for gout requiring ULT are enrolled [14]. The enrollment criteria include no history of receiving ULT within the past 4 weeks and meeting at least one of the following: two or more gout flares in a year, erosions present in radiographs of the hands or feet, or other reasons deemed necessary for ULT. This study includes data collected from September 2021 to February 28, 2024. The case report form (CRF) includes demographic and clinical data, comorbidities, lifestyle habits, medications, quality of life (as measured by the gout impact scale), the Euro-Quality of Life Five Dimension (EQ-5D) scale score, laboratory results, and radiological findings. Informed consent was obtained directly from patients on the date of registration following an in-person explanation.

All patients in this study had follow-up appointments scheduled for their next treatment and were provided with the date and time of their upcoming outpatient visit. If patients missed their scheduled appointments, no attempts were made to contact them to arrange additional visits.

The study protocol was reviewed and approved by the Institutional Review Board of Kangwon National University Hospital (approval no.:KNUH-B-2021-08-005). This study was conducted in accordance with Good Clinical Practice and the principles of the Declaration of Helsinki. Written informed consent was obtained from all participants in the ULTRA registry at the time of enrollment.

### Clinical variables of participants

We collected demographic and clinical data, including the number of attacks, comorbidities, lifestyle habits, medications, and quality of life using the Korean Gout Impact Scale (K-GIS) [15] questionnaire and the EuroQol 5-Dimension (EQ-5D) scale score, along with laboratory results and radiological findings. Data were collected on the ULTRA registry enrollment date (baseline assessment, day 0) and at follow-up (6 or 12 months later). Loss to follow-up was defined as not visiting the outpatient clinic at least once a year after initial enrollment [16–18]. Patients completed the EQ-5D to assess their health-related quality of life, which has been validated in the Korean population. The EQ-5D comprises five domains: mobility, self-care, usual activities, pain/discomfort, and anxiety/depression. Each domain is assessed using a single item with three response levels: no problems, some problems, and extreme problems. The EQ-5D score was determined using a value set developed based on the preferences of the Korean population for EQ-5D-related health conditions [19, 20].

The K-GIS is one method used to evaluate health-related quality of life (HRQOL) in patients with gout. It is the Korean version of the Gout Impact Scale, a subscale of the GAQ2.0, which has been translated and validated in Korean [15]. The K-GIS questionnaire comprises 24 items related to five distinct subscales: (1) overall gout concern (questions Q1 a-d), (2) side effects of gout medication (Q1 e, k), (3) unmet gout treatment needs (Q1 i, l, m), (4) well-being during an attack (Q2–d, Q3–g), and (5) concern during an attack (Q1 f-h, j). All K-GIS response options were given on a 5-point Likert scale (e.g., from strongly agree to strongly disagree, or from all of the time to none of the time). The K-GIS response options 1–5 were converted to a scale of 100–0 (1 = 100, 2 = 75, 3 = 50, 4 = 25, 5 = 0) for Q1 a-h, j-l, and 2 a-d, and a scale of 0–100 (1 = 0, 2 = 25, 3 = 50, 4 = 75, 5 = 100) for Q1 i, m, and 3 a-g. The five K-GIS subscale scores were calculated as the average scores of the questions included in each subscale. The overall K-GIS score was calculated as the average score of all 24 questions. A higher score indicated a more severe condition or a greater impact of gout [15, 21–24].

All patients underwent laboratory tests, including complete blood count, urate concentration, urinalysis, C-reactive protein concentration, erythrocyte sedimentation rate, serum creatinine, total cholesterol, high-density lipoprotein (HDL), low-density lipoprotein (LDL), and triglycerides at every visit.

### Statistical analysis

To compare characteristics, we calculated the frequencies and percentages for each categorical variable, and the means and standard deviations (SDs) for continuous variables. Chi-square tests and independent t-tests were used to analyze categorical and continuous variables, respectively.

Univariate logistic regression analysis was performed to identify significant risk factors for LTFU. This was used to calculate the odds ratio (OR) for LTFU, adjusting for sex, age, body mass index, comorbidities, medication, and other clinical manifestations. Comorbidities included hypertension, diabetes, dyslipidemia, ischemic heart disease, cerebrovascular disease, and chronic kidney disease. Medications included urate-lowering therapies such as allopurinol, febuxostat, and benzbromarone. Other clinical manifestations included disease duration, presence of tophi, presence of erosion on X-ray, more than two gout flares within a year, presence of malignancy, serum urate concentration, serum creatinine concentration, GIS, and EQ-5D-3L. Multivariate logistic regression analysis was conducted using forward stepwise selection on variables with a P-value of less than 0.1 in the univariate analysis. Based on the results, the ‘more than two gout flares within a year’, specific items in GIS, and EQ-5D-3L were used as variables in the multivariate logistic regression analysis.

All statistical analyses were performed using IBM SPSS Statistics for Windows, version 26.0 (IBM Corp., Armonk, NY, USA). The level of statistical significance was set at P < 0.05.

## Results

### Baseline characteristics of patients

Among the 269 patients, 144 were classified as non-LTFUs (53.5%), while 125 were identified as LTFUs (46.5%). **Table 1** presents the baseline characteristics of the study participants at registry enrollment date (baseline assessment, day 0). The mean ± SD age was 56.44 ± 17.19 years for non-LTFU patients and 54.27 ± 17.79 years for LTFU patients, showing no significant difference (P = 0.31). More than 90% of patients in both groups were men. The proportions of current smokers and drinkers were lower among non-LTFU patients compared to LTFU patients, although there was no significant difference (23.6% vs. 36.8% and 61.8% vs. 66.4%, P = 0.05 and P = 0.430, respectively). Comorbidities did not significantly differ between non-LTFU and LTFU patients: hypertension (50.0% vs. 45.6%, P = 0.47), diabetes mellitus (18.0% vs. 15.2%, P = 0.44), chronic kidney disease (11.8% vs. 10.4%, P = 0.72), malignancy (10.4% vs. 10.4%, P = 1.00), cerebrovascular disease (4.9% vs. 7.2%, P = 0.42), and ischemic heart disease (5.6% vs. 8.0%, P = 0.42).

**Table 1:** Baseline characteristics.

Variables	Non-LTFU (N = 144)	LTFU (N = 125)	P value
Age, years	56.44 ± 17.19	54.27 ± 17.79	0.310
Men	131 (91.0)	116 (92.8)	0.585
Body mass index, kg/m^2^	26.97 ± 3.85	26.46 ± 4.69	0.330
*Smoking status*			0.050
Current	34 (23.6)	46 (36.8)	
Ever	54 (37.5)	42 (33.6)	
Never	56 (38.9)	37 (29.6)	
Current alcohol consumer^a^	89 (61.8)	83 (66.4)	0.430
*Comorbidities*			
Hypertension	72 (50.0)	57 (45.6)	0.471
Diabetes	27 (18.0)	19 (15.2)	0.441
Dyslipidemia	45 (31.3)	31 (24.8)	0.241
Ischemic heart disease	8 (5.6)	10 (8.0)	0.424
Cerebrovascular disease	7 (4.9)	9 (7.2)	0.419
Chronic kidney disease	17 (11.8)	13 (10.4)	0.715
Malignancy	15 (10.4)	13 (10.4)	0.996
Disease duration, years	4.67 ± 7.19	5.66 ± 7.35	0.270
Presence of tophi	34 (25.8)	33 (27.7)	0.724
Presence of erosion on X-ray	42 (32.1)	28 (23.5)	0.133
Gout flare more than twice within a year	103 (78.0)	79 (64.8)	0.019*
*Urate-lowering therapy*			0.243
No ULT	10 (6.9)	14 (11.2)	
Allopurinol	38 (26.4)	28 (22.4)	
Febuxostat	83 (57.6)	77 (61.6)	
Benzbromarone	13 (9.0)	5 (4.0)	
Febuxostat + benzbromarone	0 (0.0)	1 (0.8)	
Serum urate concentration, mg/dL	8.79 ± 1.88	8.36 ± 1.93	0.068
Serum creatinine concentration, mg/dL	1.19 ± 0.65	1.10 ± 0.48	0.202
Total cholesterol, mg/dL	180.84 ± 45.40	181.73 ± 46.16	0.885
HDL, mg/dL	45.56 ± 12.03	45.97 ± 11.94	0.821
LDL, mg/dL	106.1 ± 42.39	111.18 ± 42.13	0.437
Triglyceride, mg/dL	215.33 ± 212.18	228.07 ± 189.61	0.677

Values are means ± standard deviations or frequencies (%).

LTFU, Loss to follow-up; ULT, Urate-lowering therapy; HLD, High-density lipoprotein; LDL, Low-density lipoprotein.

^a^Details of alcohol consumption were described in S1 Table.

*P < 0.05; **P < 0.01; ***P < 0.001.

There were no significant differences in disease duration, presence of tophi, or erosion on radiography between the two groups. A statistically significant difference was noted in the proportion of patients who experienced more than two gout flares within a year (78.0% vs. 64.8%, P = 0.02). There were no differences in the use of ULT agents between the two groups (P = 0.23).

The mean serum levels of urate, creatinine, total cholesterol, HDL, LDL, and triglycerides did not show statistically significant differences between the two groups.

### Differences of GIS and EQ-5D-3L between non-LTFU and LTFU patients

**Table 2** compares the quality of life between the non-LTFU and LTFU groups using the Gout Impact Scale (GIS) and the EQ-5D-3L. The total score of the ‘Gout concern overall’ subscale was not significantly different between the two groups (76.95 ± 19.10 for non-LTFU vs. 73.15 ± 23.33 for LTFU, P = 0.143). However, within this subscale, the item ‘Worried about a gout attack within the next year (Q1a)’ revealed a significant difference between the groups (79.17 ± 21.52 for non-LTFU vs. 73.00 ± 25.91 for LTFU, P = 0.034). The total scores of the ’Gout medication side effects’ subscale and each question were not statistically significant. The total score of the ’Gout concern during attack’ subscale was significantly higher in the LTFU group (54.30 ± 19.55 for non-LTFU vs. 59.80 ± 22.33 for LTFU, P = 0.032). In this subscale, the ’Angry during a gout attack (Q1f)’ score was higher among LTFU patients (56.25 ± 28.74 for non-LTFU vs. 63.40 ± 30.54 for LTFU, P = 0.049), as was the ’Depressive mood during a gout attack (Q1h)’ score (49.13 ± 29.03 for non-LTFU vs. 58.80 ± 30.99 for LTFU, P = 0.009). The total score of ‘Unmet gout treatment needs’ subscale and each question score did not differ significantly between the two groups. The total score of the ‘well-being during attack’ subscale was higher in LTFU patients (44.89 ± 24.72 for non-LTFU vs. 53.81 ± 25.76 for LTFU, P = 0.005). Within this subscale, the ‘Difficulty with self-care activities (Q2d)’ score was higher in LTFU patients (34.85 ± 33.12 for non-LTFU vs. 43.60 ± 34.69 for LTFU, P = 0.040). Additionally, scores for ‘Mood (Q3a),’ ‘Moving ability (Q3b),’ ‘Sleep (Q3c),’ ‘Normal work (Q3d),’ ‘Recreational activities (Q3e),’ ‘Enjoyment of life (Q3f),’ and ‘Ability to do what you want to do (Q3g)’ were significantly higher in LTFU patients compared to non-LTFU patients. Another quality-of-life indicator, the EQ-5D-3L, also demonstrated a difference between the two groups, with a higher total score in the LTFU group compared to the non-LTFU group (8.01 ± 2.48 vs. 7.11 ± 2.03, P = 0.002). Among the EQ-5D-3L questions, scores for mobility, self-care, usual activities, and pain/discomfort were significantly higher in the LTFU group than in the non-LTFU group.

**Table 2:** Differences in GIS and EQ-5D-3L between the groups with and without LTFU.

Variables	Non-LTFU (N = 144)	LTFU (N = 125)	P value
**GIS subscales**			
** Gout concern overall**	76.95 ± 19.10	73.15 ± 23.33	0.143
Q1a. Worried about a gout attack within the next year	79.17 ± 21.52	73.00 ± 25.91	0.034*
Q1b. Afraid that gout will get worse over time	76.74 ± 21.04	73.60 ± 24.86	0.263
Q1c. Anxious about gout interfering with future activities	78.99 ± 20.84	74.40 ± 25.09	0.100
Q1d. Worried about not being able to continue enjoying activities because of gout	72.92 ± 25.00	71.60 ± 26.25	0.674
** Gout medication side effects**	56.60 ± 23.46	59.00 ± 24.52	0.410
Q1e. Bothered by side effects from gout medications	52.08 ± 27.81	54.20 ± 30.24	0.551
Q1k. Worried about long-term effects of gout medication	61.11 ± 27.06	63.80 ± 25.29	0.403
** Gout concern during the attack**	54.30 ± 19.55	59.80 ± 22.33	0.032*
Q1f. Angry during a gout attack	56.25 ± 28.74	63.40 ± 30.54	0.049*
Q1g. Difficult to plan for events or activities because of gout attack	49.48 ± 26.85	51.80 ± 29.98	0.504
Q1h. Depressive mood during a gout attack.	49.13 ± 29.03	58.80 ± 30.99	0.009**
Q1j. Miss-planned important activities when gout attack	62.33 ± 28.36	65.20 ± 28.02	0.405
**Unmet gout treatment needs**	45.43 ± 13.65	44.67 ± 14.25	0.660
Q1i. Medication is effective for treating a gout attack	37.85 ± 18.22	37.40 ± 18.95	0.844
Q1l. Medication does not work well in preventing gout attack	46.53 ± 21.24	43.20 ± 19.15	0.181
Q1m. Control over gout	51.91 ± 21.13	53.40 ± 23.41	0.584
** Well‐being during attack**	44.89 ± 24.72	53.81 ± 25.76	0.005**
Q2a. Missed work due to gout symptoms	30.32 ± 35.20	34.40 ± 34.88	0.344
Q2b. Difficulty working because of gout symptoms	43.26 ± 35.21	50.60 ± 34.26	0.087
Q2c. Difficulty with recreational or social activities due to gout symptoms	45.99 ± 33.38	53.31 ± 32.90	0.078
Q2d. Difficulty with self-care activities (including feeding, bathing, and dressing) because of gout	34.85 ± 33.12	43.60 ± 34.69	0.040*
Q3a. Mood	45.26 ± 28.20	54.13 ± 32.49	0.020*
Q3b. Moving ability	55.11 ± 29.73	66.94 ± 30.31	0.002**
Q3c. Sleep	43.07 ± 33.31	54.34 ± 30.90	0.005**
Q3d. Normal work (work outside the home and housework)	49.64 ± 30.92	60.95 ± 30.09	0.003**
Q3e. Recreational activities	50.91 ± 32.00	59.09 ± 30.96	0.039*
Q3f. Enjoyment of life	48.00 ± 30.02	56.20 ± 30.50	0.031*
Q3g. Ability to do what you want to do	50.00 ± 30.16	57.64 ± 31.92	0.049*
**EQ-5D-3L total**	7.11 ± 2.03	8.01 ± 2.48	0.002**
Mobility	1.51 ± 0.56	1.70 ± 0.67	0.014*
Self-care	1.20 ± 0.44	1.34 ± 0.54	0.026*
Usual activity	1.39 ± 0.52	1.63 ± 0.63	0.001**
Pain/Discomfort	1.74 ± 0.61	1.94 ± 0.67	0.011*
Anxiety/Depression	1.27 ± 0.49	1.39 ± 0.56	0.064

Values are means ± standard deviations

GIS, Gout impact scale; EQ-5D-3L, EuroQol-5 dimension-3 level; LTFU, Loss to follow-up.

*P < 0.05; **P < 0.01; ***P < 0.001

### Logistic regression analysis for LTFU

Tables 3 and 4 present the univariate and multivariate logistic regression analyses of LTFU. In the univariate logistic regression analysis, experiencing more than two gout flares within a year was associated with a lower risk of being LTFU (OR 0.52, 95% confidence interval [CI] 0.30–0.90, P = 0.020).

**Table 3:** Univariate logistic regression analysis for LTFU.

Variables	B	OR (95% CI)	P
**Gout flares more than twice within a year**			
Not present		ref	
Present	-0.66	0.52 (0.30–0.90)	0.020*
**GIS subscales**			
**Gout concern overall**			
Q1a. Worried about a gout attack within the next year	-0.01	0.99 (0.98–1.00)	0.040*
**Gout concern during an attack**			
Q1f. Angry during a gout attack.	0.01	1.01 (1.00–1.02)	0.05
Q1h. Depressive mood during a gout attack.	0.01	1.01 (1.00–1.02)	0.010*
**Well‐being during attack**			
Q2d. Difficulty with self-care activities (feeding, bathing, dressing) because of gout symptom	0.01	1.01 (1.00–1.02)	0.040*
Q3a. Mood	0.01	1.01 (1.00–1.02)	0.020*
Q3b. Moving ability	0.01	1.01 (1.01–1.02)	0.002**
Q3c. Sleep	0.01	1.01 (1.01–1.02)	0.006**
Q3d. Normal work (work outside the home and housework)	0.01	1.01 (1.00–1.02)	0.004**
Q3e. Recreational activities	0.01	1.01 (1.00–1.02)	0.040*
Q3f. Enjoyment of life	0.01	1.01 (1.00–1.02)	0.030*
Q3g. Ability to do what you want to do	0.01	1.01 (1.00–1.02)	0.05
**EQ-5D-3L total**	0.18	1.19 (1.07–1.34)	0.002**

LTFU, Loss to follow-up; ref, reference. GIS, Gout impact scale; EuroQol-5 dimension-3 level

OR, odds ratio; CI, confidence interval.

*P < 0.05; **P < 0.01; ***P < 0.001.

**Table 4:** Multivariate logistic regression analysis for LTFU.

Variables	B	OR (95% CI)	P
**Gout flares more than twice within a year**			
Not present			
Present	-0.91	0.40 (0.22–0.75)	0.004**
**GIS subscales**			
** Gout concern overall**			
Q1a. Worried about a gout attack within the next year	-0.02	0.98 (0.97–0.99)	0.002**
** Gout concern during an attack**			
Q1f. Angry during a gout attack.	0.01	1.01 (1.00–1.02)	0.017*
Q1h. Depressive mood during a gout attack.			
** Well‐being during attack**			
Q2d. Difficulty with self-care activities (feeding, bathing, dressing) because of gout symptom			
Q3a. Mood			
Q3b. Moving ability	0.02	1.02 (1.01–1.03)	0.001**
Q3c. Sleep			
Q3d. Normal work (work outside the home and housework)			
Q3e. Recreational activities			
Q3f. Enjoyment of life			
Q3g. Ability to do what you want to do			
**EQ-5D-3L total**	0.18	1.19 (1.05–1.35)	0.007**

LTFU, Loss to follow-up; ref, reference. GIS, Gout impact scale; EuroQol-5 dimension-3 level

OR, odds ratio; CI, confidence interval. Multivariate logistic regression analysis was conducted using forward stepwise selection on variables with a P-value of less than 0.1 in the univariate analysis. Based on the results, the ‘more than two gout flares within a year’, specific items in GIS, and EQ-5D-3L were used as variables in the multivariate logistic regression analysis.

*P < 0.05; **P < 0.01; ***P < 0.001.

Within the ‘gout concern during attack’ subscale, the item ‘Depressive mood during a gout attack (Q1h)’ was also a risk factor for LTFU (OR 1.01, 95% CI 1.00–1.02, P = 0.010). In the ‘well-being during attack’ subscale, the items ‘Difficulty with self-care activities (Q2d),’ ‘Mood (Q3a),’ ‘Moving ability (Q3b),’ ‘Sleep (Q3c),’ ‘Normal work (Q3d),’ ‘Recreational activities (Q3e),’ and ‘Enjoyment of life (Q3f)’ were risk factors for LTFU. A higher EQ-5D-3L total score was associated with an increased risk of LTFU (OR 1.19, 95% CI 1.07–1.34, P = 0.002). The multivariate logistic regression analysis revealed that ‘Angry during a gout attack (Q1f)’ (OR 1.01, 95% CI 1.00–1.02, P = 0.017), ‘Moving ability (Q3b)’ (OR 1.02, 95% CI 1.01–1.03, P = 0.001), and EQ-5D-3L total score (OR 1.19, 95% CI 1.05–1.35, P = 0.007) were associated with an increased risk of LTFU. Conversely, experiencing more than two gout flares within a year (OR 0.40, 95% CI 0.22–0.75, P = 0.004) and scores for ‘Worried about a gout attack within the next year (Q1a)’ were associated with a decreased risk of LTFU (OR 0.98, 95% CI 0.97–0.99, P = 0.002) showing a similar trend to the results of the univariate regression analysis.

## Discussion

This study identified several risk factors associated with LTFU using prospective cohort data on gout. The results indicated that greater anger experienced and more significant limitations in mobility during a gout attack correlated with a higher risk of LTFU. Additionally, higher scores on the EQ-5D-3L scale and other quality-of-life measures were associated with an increased risk of LTFU. However, experiencing more than two flare-ups within a year and concerns about a gout attack in the following year were linked to a decreased risk of LTFU.

Based on previous research, adherence rates for other rheumatic diseases have been reported as follows: Rheumatoid arthritis (RA) has an adherence rate ranging from 30% to 98%, systemic lupus erythematosus (SLE) from 48% to 92%, juvenile Idiopathic Arthritis (JIA) from 86% to 92%, and polymyalgia rheumatica (PMR) approximately 88% [5, 25–39]. In comparison, patients with gout demonstrated relatively low adherence rates, ranging from 18% to 74% [25].

De Klerk et al. monitored 12 patients on colchicine and 17 on urate-lowering agents for 12 months, finding adherence rates of 44% for colchicine and 74% for urate-lowering agents based on pill bottle openings [5, 25]. Sarawate et al. discovered that only 26% of 2,405 allopurinol users had a medication possession ratio (MPR) of 80% or higher, with a median treatment duration of three months [6, 25]. Riedel et al. analyzed adherence in 5,597 allopurinol users over 24 months and found that only 18% maintained an MPR of 80% or higher, with an average adherence rate of 56% during the treatment period [4, 25].

These studies highlight the challenges in achieving high adherence rates for gout treatment, with a significant proportion of patients failing to consistently follow prescribed medication regimens. While active research exists on drug adherence in gout treatment, only a few studies have focused on LTFU.

In a similar study on LTFU in gout, older age was associated with a higher follow-up rate, and patients with more severe gout, characterized by polyarticular disease, also exhibited better follow-up rates in multivariate analysis [12]. However, in this study, no significant differences in LTFU were observed based on age. Patients experiencing more than two gout flares per year had a significantly higher follow-up rate, indicating a trend consistent with previous studies. Unlike earlier research, this study revealed new insights into how the emotional state of patients with gout influences LTFU.

Gout is a chronic disease that requires consistent management. Without appropriate maintenance treatment, patients may endure severe pain due to gouty arthritis and face an increased risk of various complications [9]. Previous studies have indicated that, despite being a chronic condition, patients with gout often experience inadequate follow-up or poor adherence, an issue prevalent in daily clinical practice [9, 40–43].

A common perception among the public is that gout is a condition that necessitates treatment only during painful episodes. Additionally, the intercritical periods between attacks can be prolonged, leading to a failure to recognize it as a condition requiring ongoing treatment [41–43]. Another contributing factor to high LTFU rates is the nature of gout, where pain typically resolves relatively quickly with medications such as anti-inflammatory drugs and steroids [44]. These factors collectively reduce regular follow-up rates. If follow-ups are not conducted regularly and gout is not managed effectively, it can lead to joint damage, frequent attacks, and a severely impaired quality of life [44].

Our results indicated that patients who experienced more than two flares annually and those expressing concern about a gout attack within the next year had a lower risk of LTFU.

One possible hypothesis for the reduced risk of lost to follow-up (LTFU) associated with gout flares more than twice within a year is that experiencing frequent flares may prompt these individuals to recognize the necessity for treatment, ultimately lowering their risk of LTFU. In contrast, those with few or no gout attacks, or those who have long intercritical periods, may perceive less need for ongoing treatment, which could contribute to an increased likelihood of becoming lost to follow-up.

Patients who were worried about future gout attacks were more likely to visit hospitals regularly and adhere to their treatment plans. In contrast, negative emotions, such as anger, appeared to increase the risk of LTFU. This study demonstrated that, similar to hypertension and other chronic conditions, gout is negatively affected by emotions like depression and anger in relation to LTFU.

Some studies have reported that emotional factors, including anxiety, depression, and anger, contribute to noncompliance in other chronic diseases. Mild anxiety and depressive symptoms have been linked to increased risk of nonadherence to antihypertensive medications in patients with hypertension [45]. DiMatteo et al. also found that depressed patients were three times more likely to be noncompliant with medical treatment than nondepressed patients, with an odds ratio of 3.03 (95% CI, 1.96–4.89) [46].

Multivariate analysis revealed that anger experienced during a gout attack was a significant risk factor for LTFU in this study for the first time. These results appear to align with the emotional influences on compliance seen in other chronic diseases; however, they also highlight differences by presenting distinct risk factors compared to those previously studied in relation to medication adherence among gout patients. The previously identified risk factors that reduced medication adherence were younger age, male gender, and fewer comorbidities, which differ from the emotional factors highlighted in this study.

Emotional changes associated with depression, such as sadness or anger, can hinder necessary follow-up for managing chronic conditions and diminish patient compliance, leading to inconsistent treatment and care. Research has also indicated a higher risk of depression among patients with gout [47, 48]. Similarly, depressive symptoms can pose challenges for patients with gout in maintaining consistent follow-up, potentially resulting in higher treatment discontinuation rates. This can lead to more frequent gout attacks and overall poorer disease management [47–49].

Therefore, appropriate interventions for patients experiencing emotions such as depression or anger may improve follow-up rates and ultimately facilitate effective gout management. Ma et al. demonstrated that cellphone-based chat interventions can assist in managing depression and emotions in patients with chronic conditions, thereby improving follow-up care for these conditions [50]. Interventions utilizing accessible methods can enhance follow-up rates by effectively addressing emotional concerns. These interventions need not be cellphone-based; various other methods could be employed. For example, surveys assessing emotional stress related to gout and quality of life could be conducted during initial hospital visits. By identifying the extent of depression-related factors and other negative emotional stressors, proper education and emotional support can be provided. It is reasonable to anticipate improved follow-up rates during subsequent visits.

In this study, patients who perceived mobility limitations exhibited a higher rate of LTFU. Based on these findings, comprehensive education should be provided during initial visits to individuals identified with these factors. This education should cover how to use assistive devices, such as canes, walkers, or wheelchairs, and emphasize the importance of regular follow-up and prevention of gout flare [11, 40]. Furthermore, health professionals should be able to identify where assistive devices are needed and it seems necessary to provide these devices. The EQ-5D-3L total score reflects overall quality of life, with higher values indicating poorer quality of life. In this study, the EQ-5D-3L total score was identified as a risk factor for LTFU, and it can be viewed as similar to the findings regarding specific items from the K-GIS, another measure of quality of life. The EQ-5D-3L consists of five domains (mobility, self-care, usual activities, pain/discomfort, and anxiety/depression), and regression analysis revealed that the total score, which combines all the domains, rather than individual items, was associated with LTFU. This distinction from the K-GIS, which showed significant results for specific items, may stem from differences in the content and structure of the questionnaires.

There are several limitations of this study. There is a possibility that unmeasured confounding factors were not accounted for in the model because this is an observational study. Additionally, the study’s case report form does collect information on mortality and causes of death; however, if a participant died outside the hospital, or they moved to a different clinic, we could not obtain the exact data. It can be confounding factors in the analysis. It seems that a more sophisticated tool is needed for evaluating and recording these factors in future research.

Some risk factors exhibited modest OR, which may not strongly support claims of a high risk of LTFU for these items. However, despite their relatively lower odds ratios, multivariate regression analysis confirmed significant differences in these factors, suggesting a degree of credibility. Similar items included in the analysis demonstrated comparable risks, thus supporting their reliability.

This study identified risk factors for LTFU in patients with gout, underscoring their importance in predicting LTFU through an assessment of initial visit evaluation factors. These factors include emotional states such as depression, anger, and mobility issues, which directly impact daily life. Recognizing these factors during the initial visit may aid in targeting efforts to reduce LTFU. Based on these findings, it is crucial to pay closer attention to patients who report being affected by mood changes in initial surveys and to provide them with appropriate education and emotional support. Offering guidance and encouragement for regular follow-ups can reduce LTFU in at-risk individuals, ultimately enhancing the overall management of gout.

## Supporting information

S1 Table Type of alcohol consumption and amount in current alcohol consumers. (DOCX)
