# Supplementary material for: Risk factors for loss to follow-up in patients with gout: A Korean prospective cohort study
Source: PLoS One. 2025 Feb 7;20(2):e0318564. doi: 10.1371/journal.pone.0318564 (PMC11805391; doi:10.1371/journal.pone.0318564)
Supplement: S1 Table — (DOCX) [file pone.0318564.s001.docx]

| Variables | Non-LTFU (N = 89) | | LTFU (N = 83) | | P value | | Missing value | |
| --- | --- | --- | --- | --- | --- | --- | --- | --- |
| **Type of alcohol consumption** |  | |  | | 0.432 | | 0 (0) | |
| Beer | 12 (13.5) | | 10 (12.0) | |  | |  | |
| Korean distilled spirit (Soju) | 75 (84.3) | | 69 (83.1) | |  | |  | |
| Korean rice wine (Makgeolli) | 1 (1.1) | | 2 (2.4) | |  | |  | |
| Whiskey | 0 | | 1 (1.2) | |  | |  | |
| All kind of alcohol | 1 (1.1) | | 1 (1.2) | |  | |  | |
| Others | 0 | | 0 | |  | |  | |
| **Frequency of drinking** |  | |  | | 0.132 | | 1 (0.59) | |
| Less than once a week | 31 (35.2) | | 22 (26.5) | |  | |  | |
| 1-2 times a week | 30 (34.1) | | 25 (30.1) | |  | |  | |
| 3-4 times a week | 13 (14.8) | | 20 (24.1) | |  | |  | |
| 5 or more times a week | 14 (15.9) | | 16 (19.3) | |  | |  | |
| **Amount of alcohol per drinking**  (One glass of beer 225cc, One glass of soju 75cc, |  | |  | | 0.074 | | 0 (0) | |
| One glass of wine 125cc, One bottle of beer 640 ml,  One bottle of soju 360ml, One bottle of wine 750ml) |  | |  | |  | |  | |
| 1 glass | 5 (5.6) | | 2 (2.4) | |  | |  | |
| 2 glasses | 6 (6.7) | | 4 (4.8) | |  | |  | |
| 3 glasses | 8 (9.0) | | 2 (2.4) | |  | |  | |
| 4 glasses - 1 bottle | 22 (24.7) | | 16 (19.3) | |  | |  | |
| 1-2 bottle | 33 (37.1) | | 48 (57.8) | |  | |  | |
| More than 2 bottles | 15 (16.9) | | 11 (13.3) | |  | |  | |
| Values are frequencies (%).  LTFU, Loss to follow-up.  *P < 0.05; **P < 0.01; ***P < 0.001 | |  | |  | |  | |  |

**S1 Table. Type of alcohol consumption and amount in current alcohol consumers**
